# Supplementary figures and images for: Metabolomic Analysis Identifies Betaine as a Key Mediator of TAp73α-Induced Ferroptosis in Ovarian Granulosa Cells
Source: Int J Mol Sci. 2025 Jun 24;26(13):6045. doi: 10.3390/ijms26136045 (PMC12250254; doi:10.3390/ijms26136045)

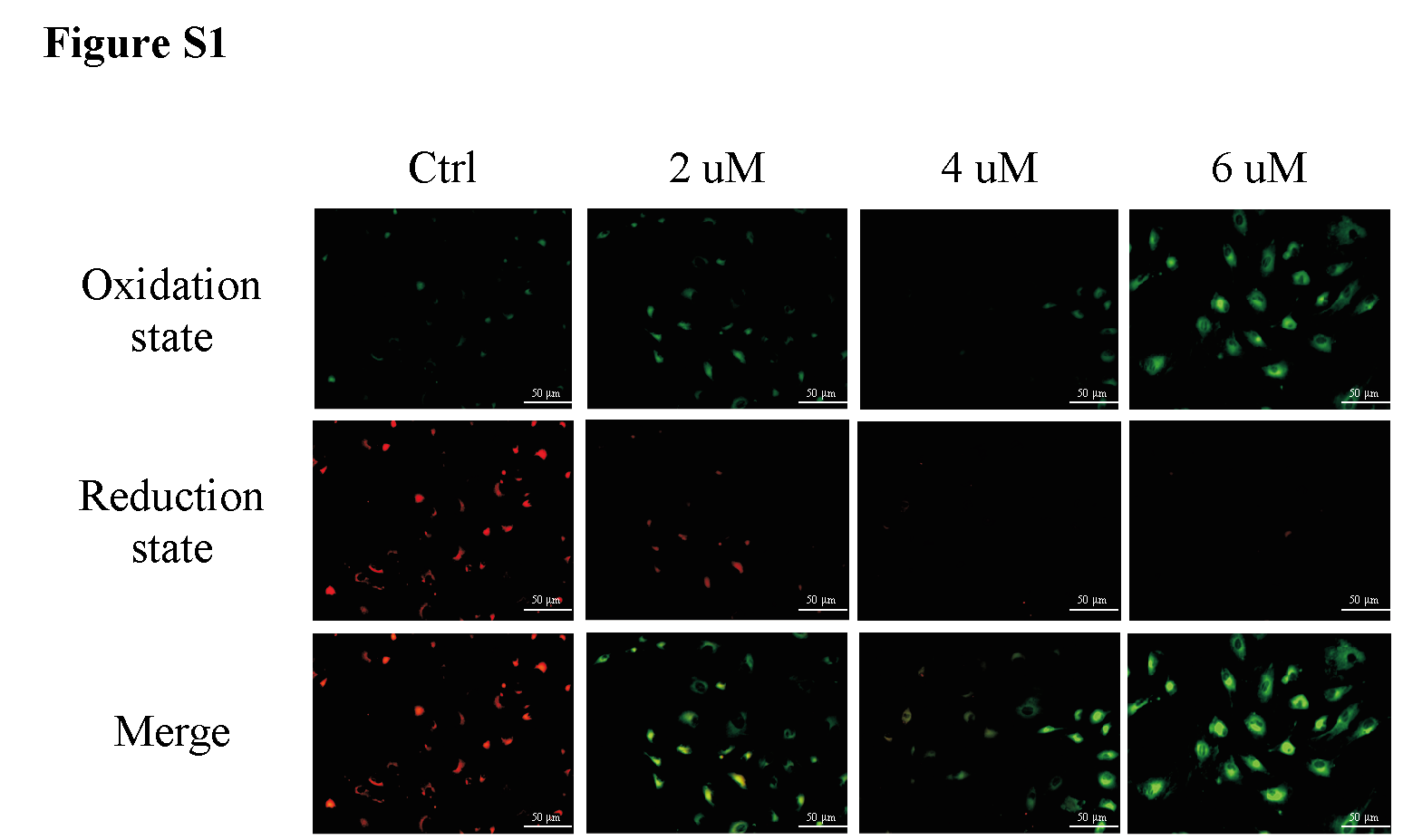

Supplement: Supplementary file 1 [file ijms-26-06045-s001.zip › Figure S1 Cattle follicular granulosa cells can undergo ferroptosis.tif]
